# First-pass myocardial stress perfusion MRI using k-t BLAST at an open MR-system: initial results in comparison to FFR measurements

**DOI:** 10.1186/1532-429X-15-S1-P184

**Published:** 2013-01-30

**Authors:** Ortrud Kosiek, Katharina A Strach, Bernhard Schnackenburg, Alexander Schmeisser, Jan Smid, Friederike Walz, Jens Ricke, Frank Fischbach

**Affiliations:** 1 Department of Radiology and Nuclear Medicine, University Magdeburg, Magdeburg, Germany; 2 Department of Cardiology, University Magdeburg, Magdeburg, Germany; 3 Clinical Science, Philips Healthcare, Hamburg, Germany

## Background

First-pass myocardial stress perfusion MR imaging (MRI) is routinely used for the detection of myocardial ischemia in patients with suspected or known coronary artery disease (CAD). Up to now, cardiac MRI stress testing had to be performed in a closed bore environment. To allow for MR stress perfusion in claustrophobic patients and to improve monitoring during stress examination and in critically ill patients an open MR platform seems desirable. Recently, an open 1.0T MR-system with high gradient performance has been introduced, which may allow for first-pass myocardial stress perfusion in combination with fast data acquisition techniques such as k-t BLAST.

**Figure 1:**
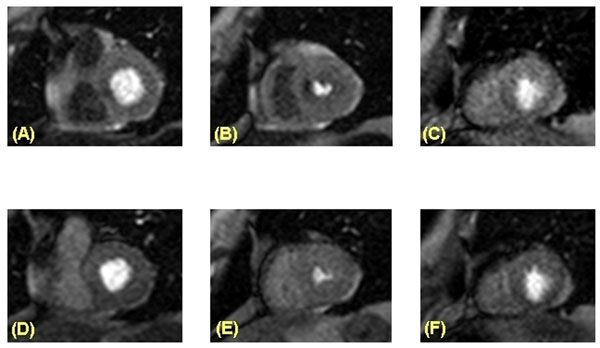
Adenosine stress perfusion (stress study A-C, rest study D-F) MRI in a 81 year old patient with increasing dyspnoea. Stress-induced perfusion deficits septal in the basal (A) and mid-ventricular (B) segments without correlate during resting conditions (D-F). Conventional angiography revealed a hemodynamically relevant (fractional flow reserve <0.8) stenosis in a septal coronary artery branch.

Therefore, the aim of our study was to evaluate 1) feasibility of adenosine stress perfusion (ASP) MRI using k-t BLAST in patients with suspected or known CAD in a clinical setting as well as 2) Contrast Enhancement Ratio (CER) and image quality at an open MR system.

## Methods

58 patients (18 female, 40 male; 62+/-13 years) with known or suspected CAD underwent a standard ASP at an open Panorama HFO 1.0 Tesla System (Philips Healthcare, Best, Netherlands). First-pass perfusion was acquired using a k-space segmented T1-weighted gradient-echo sequence (in-plane resolution 3x3 mm2, slice thickness 10 mm, k-t factor 3.5) at rest and during i.v. adenosine administration (140 µg/kg BW for 4 minutes). The CER (=myocardial peak signal - myocardial baseline signal/myocardial baseline signal) for stress perfusion studies and overall image quality (4-point grading scale: 4: excellent; 1: non-diagnostic) both of rest and stress examinations were assessed. A semiquantitative analysis of dark rim artifacts was performed and transmural extent (1:<25%; 2: 25-49%; 3:50-74%; 4: 75-100%) evaluated. Hemodynamically significant CAD was defined as fractional flow reserve <0.8 using conventional coronary angiography.

## Results

All examinations (n=58/58) were completed successfully. CER of stress perfusion was 2.52+/-1.14. Overall image quality was 3.37+/-0.67 for rest and 3.54+/-0.66 for stress perfusion. Dark rim artefacts appeared in 129 of 928 slices (14%) and were predominately subendocardial (1:93/129, 72%; 2:15/129, 12%; 3:12/129, 9%; 4:9/129, 7%). Sensitivity and specificity for detection of significant CAD was 91% and 82%, respectively.

## Conclusions

First-pass stress perfusion MR imaging at an open 1.0 T MR-system is feasible in a clinical setting and yields promising results for the detection of significant coronary artery stenosis. Additionally, using k-t BLAST ASP provides good image quality and contrast, while preserving good temporal and spatial resolution.

## Funding

None.

